# Deep-WET: a deep learning-based approach for predicting DNA-binding proteins using word embedding techniques with weighted features

**DOI:** 10.1038/s41598-024-52653-9

**Published:** 2024-02-05

**Authors:** S. M. Hasan Mahmud, Kah Ong Michael Goh, Md. Faruk Hosen, Dip Nandi, Watshara Shoombuatong

**Affiliations:** 1 Department of Computer Science, American International University-Bangladesh (AIUB), Kuratoli, Dhaka, 1229 Bangladesh; 2 Centre for Advanced Machine Learning and Applications (CAMLAs), Dhaka, 1229 Bangladesh; 3 Faculty of Information Science & Technology (FIST), Multimedia University, Jalan Ayer Keroh Lama, 75450 Melaka, Malaysia; 4 Department of Information and Communication Technology, Mawlana Bhashani Science and Technology University, Santosh, Tangail, 1902 Bangladesh; 5 Center for Research Innovation and Biomedical Informatics, Faculty of Medical Technology, Mahidol University, Bangkok, 10700 Thailand

**Keywords:** Computational models, Biological techniques, Computational biology and bioinformatics

## Abstract

DNA-binding proteins (DBPs) play a significant role in all phases of genetic processes, including DNA recombination, repair, and modification. They are often utilized in drug discovery as fundamental elements of steroids, antibiotics, and anticancer drugs. Predicting them poses the most challenging task in proteomics research. Conventional experimental methods for DBP identification are costly and sometimes biased toward prediction. Therefore, developing powerful computational methods that can accurately and rapidly identify DBPs from sequence information is an urgent need. In this study, we propose a novel deep learning-based method called Deep-WET to accurately identify DBPs from primary sequence information. In Deep-WET, we employed three powerful feature encoding schemes containing Global Vectors, Word2Vec, and fastText to encode the protein sequence. Subsequently, these three features were sequentially combined and weighted using the weights obtained from the elements learned through the differential evolution (DE) algorithm. To enhance the predictive performance of Deep-WET, we applied the SHapley Additive exPlanations approach to remove irrelevant features. Finally, the optimal feature subset was input into convolutional neural networks to construct the Deep-WET predictor. Both cross-validation and independent tests indicated that Deep-WET achieved superior predictive performance compared to conventional machine learning classifiers. In addition, in extensive independent test, Deep-WET was effective and outperformed than several state-of-the-art methods for DBP prediction, with accuracy of 78.08%, MCC of 0.559, and AUC of 0.805. This superior performance shows that Deep-WET has a tremendous predictive capacity to predict DBPs. The web server of Deep-WET and curated datasets in this study are available at https://deepwet-dna.monarcatechnical.com/. The proposed Deep-WET is anticipated to serve the community-wide effort for large-scale identification of potential DBPs.

## Introduction

DNA-binding Proteins (DBPs) participate in many essential biological processes, including DNA replication, gene regulation, repair, and modification^1,2^. Identification of DBPs is fundamentally important for understanding characterizations of protein function and drug design. A number of large-scale proteomics experiments have been performed to identify DBPs based on biochemical methods, such as X-ray crystallography^3^ and fast ChIP^4,5^. Despite the increasing number of experimentally determined DBPs, the underlying mechanism of DBP specificity remains mostly unidentified, and these approaches are laborious, time-consuming, and sometimes biased toward prediction in the post-genome era, when large numbers of unannotated DBPs are rapidly being sequenced and deposited. As an alternative, computational methods are accurate and cost-effective and can be used to complement the experimental efforts.

To date, several computational algorithms, including machine-learning (ML)-based and template-based methods, have been developed for in silico prediction of DBPs^6–17^. DBPs can be predicted based on two types of protein data input: i sequence-driven (e.g., iDNA-Prot^18^, PseDNA-Pro^19^, iDNAPro-PseAAC^20^, iDNA-Prot|dis^14^, Local-DPP^21^, PSFM-DBT^22^, HMMBinder^23^, IKP-DBPPred^24^, iDNAProt-ES^7^, DPPPseAAC^10^, and TargetDBP^25^) and 3D-structure-driven (e.g., DBD-Hunter^26^, iDBPs^27^, and SPOT-Seq (DNA)^28^) methods. Only protein sequence data is required for sequence-driven techniques. The 3D-structure-driven techniques require native or projected 3D structure data from the query protein. In this case, 3D-structure-driven techniques cannot function correctly without 3D structure information. This method performs better when the protein’s native structure is known. On the other hand, sequence-driven techniques do not have this problem. Furthermore, due to the inherent challenges of measuring protein 3D structures in experimental studies, there is a significant gap between the quantities of sequences and 3D structures^29^, which is currently expanding quickly in the postgenomic era. Recently, PSI-BLAST was utilized by Chowdhury et al.^7^ to derive polysaccharide storage myopathy, which revealed evolutionary information to predict DBP. The secondary structure information of the protein sequences was extracted using SPIDER2. To retrieve protein sequence information, Nanni et al.^30^ utilized AAC and quasi residue couple (QRC). Meanwhile, the autocovariance method was used to derive physicochemical characteristics. In addition, evolutionary data was retrieved using the pseudo-position specific scoring matrix (PsePSSM), N-gram features (NGR), and texture descriptors (TD). Sang et al.^31^ calculated the HMM matrix for each sequence using the hidden Markov model (HMM). The HMM matrix was converted into feature vectors of the same length using AAC, autocovariance transformation (ACT), and cross-covariance transformation (CCT). Thus, designing sequence-driven computational strategies is essential to accurate prediction of DBPs.

Choosing appropriate feature extraction methods and classification algorithms in order to select the best subset of features is a key factor for the successful discovery of DBPs. In TargetDBP^25^, four single-view features (AAC, PsePSSM, PsePRSA, and PsePPDBS) are used to extract the DNA-binding features and apply a learning-based technique to the weights of features to combine them for training an SVM classifier. In addition, an excellent feature subset was selected using SVM-REF + CBR from the non-redundant benchmark and new gold-standard dataset. Rahman et al.^10^ utilized the same feature selection (REF) and classifier (SVM) to develop a model DPP-PseAAC for which the authors focused on Chou’s general PseAAC for generating features. Another proposed DBP predictor method is DNAPred^32^, where authors use the E-HDSVM algorithm, which includes HD-US and EAdaBoost, to predict protein DNA binding sites. A similar ensemble-based method performed by Zhang et al.^33^, XGB-RFE, is used to attain effective features, after which the best features are fed to the stacked ensemble classifier (the combined form of LightGBM, XGBoost, and SVM) to build the proposed StackPDB model.

The above-mentioned algorithms have proven to be exemplary, but we opted to use convolutional neural networks (CNNs) to improve prediction performance. In the meanwhile, it is important to devise an appropriate encoding scheme to represent the sequence fragments surrounding DBPs/non-DBPs to develop a ML-based predictor. In this study, we present a new convolutional neural network (CNN)-based predictor called Deep-WET for accurately identifying DBPs from primary sequence information. Firstly, we applied three consecutive sequence encoding approaches, namely Global Vectors (GloVe), Word2Vec, and fastText, to extract the protein sequence patterns. Secondly, the DE is utilized to acquire the weights for three base features. With these obtained weights, we combined three base features in a weighted manner to create the super feature. In order to improve the predictive performance of Deep-WET, we employed SHapley Additive exPlanations (SHAP) approach to remove irrelevant features from super features and then inputted the optimal one into CNN algorithm for the final model construction. Experimental results demonstrated that Deep-WET achieved a accurate and robust performance as compared with conventional ML classifiers on both the training and independent test datasets. Moreover, comparative analysis on the independent test dataset showed that Deep-WET achieved improved performance compared with the existing approaches, highlight the effectiveness and robustness of the proposed Deep-WET. We also conducted a series of computational analyses to provide in-depth understanding of the DBPs. Finally, the proposed method, Deep-WET, was implemented as a user-friendly web server: at https://deepwet-dna.monarcatechnical.com/.

## Materials and methods

### The overall framework of Deep-WET

**Figure 1:**
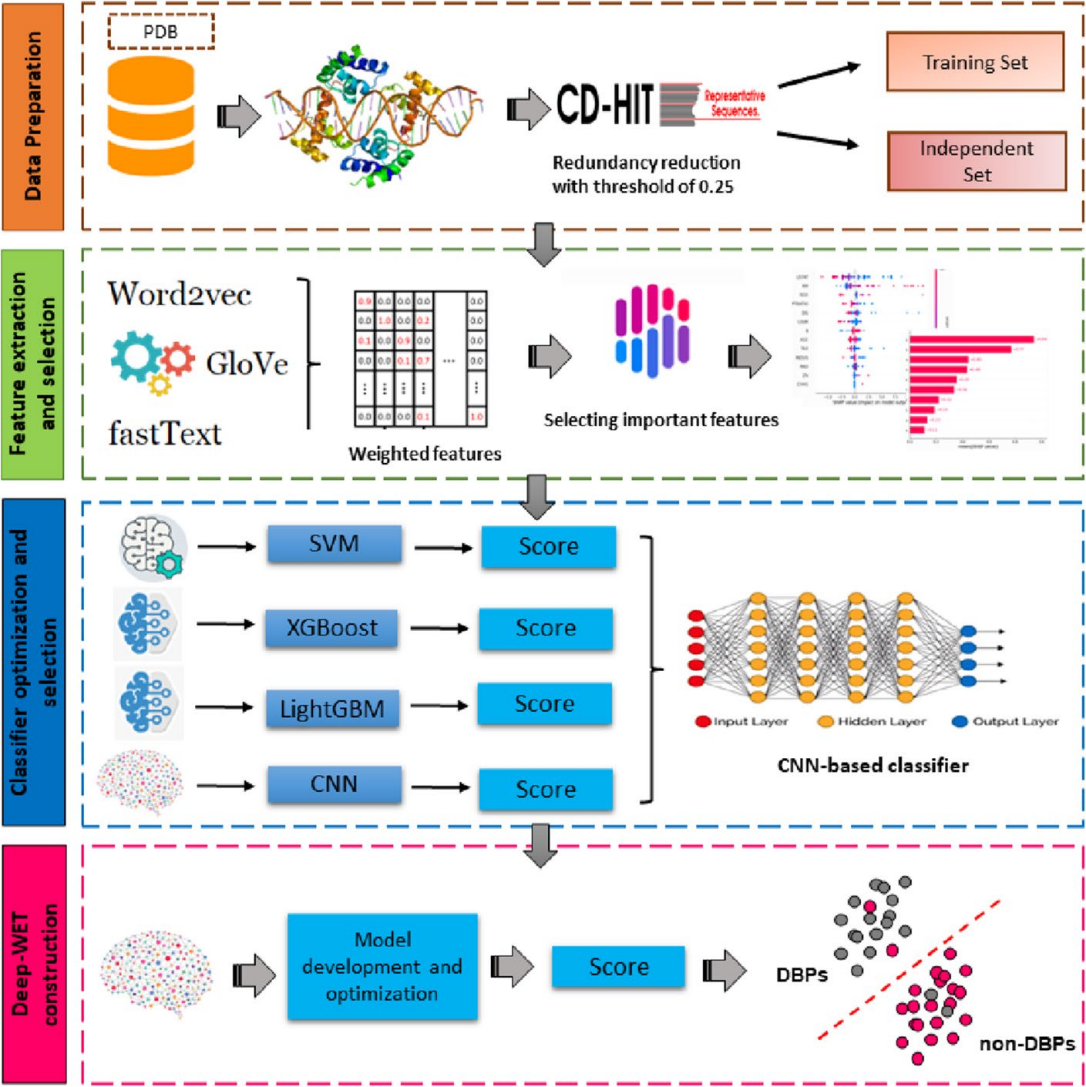
The flowchart illustrates our proposed methodology. The upper part represents data pre-processing, the middle part depicts feature extraction with various classifiers, and the lower part showcases classification using the CNN model.

The construction process of Deep-WET is depicted in Fig. 1. Deep-WET consists of multiple steps, including data preparation, natural language processing (NLP)-based feature encoding, weighted features, optimal feature subset selection, best classifier selection, and final prediction. In the first stage, three NLP-based Word embedding feature encoding techniques were employed (GloVe, Word2Vec, and fastText), and then the optimal subset of features was selected using the SHAP technique from the weighted features. The selected feature subsets from each feature encoding were fed to four ML and one DL algorithms to build the final prediction models using the training and independent test datasets. Finally, the classifier having the highest cross-validation AUC was considered to construct the final predictor herein.

### Data preparation

Developing a reliable, comprehensive, and stringent dataset is the first important step of statistical predictor development. Here, the curated dataset denoted with S was presented as:

1 $$\begin{aligned} S = S_{posi} \cup S_{nega} \end{aligned}$$

where, $S_{posi}$ denotes the positive subset containing DBPs or positive samples, while, $S_{nega}$ denotes the negative subset containing non-DBPs or negative samples, and $\cup$ symbol resembles the union of the following sets. The $S_{posi}$ and $S_{nega}$ datasets were collected and primarily used by Jun Hu et al.^25^, who collected both DBP and non-DBP chains from PDB Data Bank. There are two main reasons why we used the dataset established by Jun Hu et al.^25^ as follows. Firstly, this dataset applied a lower CD-HIT^34^ threshold of 0.25 to exclude the redundant protein chains. Secondly, this dataset exclude the protein chain sequences having below 50 residues and unknown residues. For the the $S_{posi}$ and $S_{nega}$ datasets, they were randomly selected to create the training and independent test datasets. The training dataset consists of 1052 DBPs and 1052 non-DBPs, while The independent test dataset consists of 148 DBPs and 148 non-DBPs. More details on the training and independent test datasets are provided in an article of Jun Hu et al.^25^.

### Feature encodings

Word embedding (WE), in which the vocabulary of words can be represented as vectors using large text as an input, is the most popular technique in the area of natural language processing (NLP). WE techniques are able to convert amino acids in a fixed-length vector, where a user needs to define the fixed feature dimensions that can provide adequate prediction results. In this study, we implemented three unsupervised embedding techniques to encode protein sequences: GloVe^35^, Word2Vec^36^, and fastText^37–39^.

#### Word2Vec

Word2Vec, a model developed by Tomas Mikolav at Google, computes and generates high-quality, distributed, and continuous dense representations of words^36^. These are unsupervised models that can take in massive textual corpora, create a vocabulary of possible word combinations, and generate dense word embeddings on the vector space. The size of the vocabulary determines the size of the word embedding vectors. This decreases the dimensionality of the following dense vector, compared to high-dimensional sparse vector generation using the traditional bag of words (BOW). To construct word embedding, Word2Vec employs two different methods: (1) common bag of words (CBOW) and (2) the Skip-gram model. Notably, the CBOW is faster than the Skip-gram model and generates a better representation of more frequent words^34^. On the other hand, the Skip-gram model performs well with a relatively small amount of data and generates a better representation of rare words^34^.

Finding the target word $w_t$ through n predictions using the CBOW model can be accomplished by the following equation:

2 $$\begin{aligned} J_{\phi } = \frac{1}{T}\sum _{t=1}^{T}logP(w_t \big | w_{(t-n)}, \ldots ,w_{(t-1)},w_{(t+1)}, \ldots ,w_{(t+n)}) \end{aligned}$$

Here, $w_{(t-1)}$ to $w_{(t+n)}$ sequence of words represents the context words. The following equation can further simplify the above equation since the hidden layer can be equivalent to a softmax layer:

3 $$\begin{aligned} P(w_t \big | w_{(t-n)}, \ldots ,w_{(t-1)},w_{(t+1)}, \ldots ,w_{(t+n)}) = \frac{exp(W_k^Th_t)}{\sum _{k=1}^{v} exp(W_k^Th_t)} \end{aligned}$$

Here, the output weight matrix between hidden layers is denoted as *W*, and after matrix operation, the average value of input vectors is represented as $h_t$.

#### GloVe

GloVe is an unsupervised learning vectorization technique. It is a log-bilinear regression model that incorporates both local statistics and global statistics^36^. The training of this model is performed on non-zero entries of global word-to-word co-occurrence statistics that tabulates how frequently words are co-occurring within a given corpus. For collecting statistics, the following matrix needs a single pass through the entire corpus. These passes can be expensive for large corpora. Moreover, its resulting representations show the interesting linear substructures of those word vector spaces.

4 $$\begin{aligned} \sum _{i,j}^{N}f(X_{ij})(v_i^Tv_j + b_i + b_j - log(X_{ij}))^2 \end{aligned}$$

Here, $v_i, v_j$ correspond to the word embedding of *i*, *j*; *X* represents the word-to-word co-occurrence matrix; and $i^{th}$ number of co-occurrences of word *j* is denoted by $X_{ij}$. Furthermore, the probability of word *j* occurring in the context *i* is the following:

5 $$\begin{aligned} X_{ij} = P(i) = \frac{X_{ij}}{X_i} \end{aligned}$$

#### fastText

fastText, proposed by Facebook^38^, is an extension of Word2Vec. It provides tools to learn word representation and sentence classifications of ML. Word vectors are a more organized, numerical, and efficient representation of words and sentences. fastText provides a supervised module to build a model for text classifications. It technique breaks an individual word into a bag of n-grams or sub-words and feeds them into the network, which also generates vector representation for rare or unseen words^37^. Since the technique uses the same architecture as Word2Vec, the following equation minimizes the loss of softmax layer, *l* over *N* sequences using CBOW model:

6 $$\begin{aligned} \sum _{n=1}^{n} l(y_n, BAx_n) \end{aligned}$$

Here, $x_n$ represents the bag of one-hot encoded vectors and $y_n$ represents the label of the nth sequence of words. The purpose of using FastText in the present study is to find the partial information single DNA sequence order.

### Weight learning for weighted features

Single-view features represent the discriminative information for each sequence, but combing single-view features to make a weighted feature is critical in ML-based DBP prediction. The most common technique involves serially adding (’+’) single features. However, this straightforward combination technique lacks a guarantee to represent discriminative capability and may overlook the relative importance of the base sequence. To address this issue, we employ a differential evolution (DE) method to determine the optimal weights for each feature. DE algorithm variants of evolutionary algorithms and applied in various works^40,41^ to show the positive effect. The process we followed for DE algorithm to learn feature weights from a single feature is illustrated as follows:

**Step 1:** **Initialization** Randomly create an initial population $P_o = \{FW_1^g, FW_2^g, FW_3^g \ldots \, \ldots \,FW_n^g\}, where (FW_{i,1}^g, FW_{i,2}^g, FW_{i,3}^g, FW_{i,4}^g)^T$ represents *i*th number solution in the population *g*th. *N* means size of the generation population where to set the maximum generation $G_{max}$, crossover rate (*CR*), scaling factor (*F*) to 1000, 0.5, and 0.5, respectively.
**Step 2:** **Mutation** A mutation vector $MV_i^g$ was initialized for each salutation according.
  7 $$\begin{aligned} MV_i^g = FW_{r1}^g + F.(FW_{r2}^g - FW_{r3}^g) \end{aligned}$$
**Step 3:** **Crossover** For the diversity of each solution, a trial vector $TV_i^g = (TV_{i,1}^g, TV_{i,2}^g, TV_{i,3}^g \ldots \, \ldots \,TV_{i,D}^g)$ of crossover is established in the DE technique as follows:
  8 $$\begin{aligned} TV_{i, j}^g = {\left\{ \begin{array}{ll} &{} \text {TV}_{i,j}^g \quad if \; R_j \le CR \;\;ot \;j = j_r\\ &{} \,\,\text {FW}_{i,j}^g \qquad \,\,\text {otherwise}\\ \end{array}\right. } \end{aligned}$$
  where $j = 1,2,3 \ldots ,D, j_r$ represent randomly produced integer with [1, *D*]; $R_j$ means uniformly distributed range [0,1] and $CR \in (0,1)$ indicate crossover rate.
**Step 4:** **Selection** Find the better vector from trial $TV_i^g$ and target $FW_i^g$ using the following way:
  9 $$\begin{aligned} FW_i^{g+1} = {\left\{ \begin{array}{ll} &{} TV_{i}^g, \quad \text {if} \; f(TV_i^{g}) \le f(FW_i^g)\\ &{} \,\,\text {FW}_{i}^g, \qquad \,\,\text {otherwise}\\ \end{array}\right. } \end{aligned}$$
**Step 5:** **Termination** $g=g+1$ and repeat steps 2 to 4 until *g* is greater the $G_{max}.$

After concluding the DE procedure, we can get the final results. In this study, We have generated a novel super feature, represented as GloVe + fastText + Word2Vec, by the weighted and sequential fusion of GloVe, fastText, and Word2Vec features. DE is a powerful optimization algorithm; however, using it for feature weighting in ML presents certain limitations and challenges. DE may struggle with slow convergence, susceptibility to local optima, and sensitivity to parameter choices. Additionally, the algorithm may violate constraints, lack robustness across diverse datasets, and exhibit computational intensity. To avoid these challenges, we have performed parameter tuning $(population-size, \, mutation \, rate, \, crossover \, probabilities)$ in experiments, considering adaptive strategies for mutation and crossover rates. Furthermore, exploring parallelization methods helps alleviate computational burdens, while strategies like diversity maintenance mechanisms aim to address convergence issues.

### SHAP-based feature selection scheme

SHAP is an additive feature attribution method introduced by Lunberg and Lee^42^ in which each individual prediction is interpreted by the contribution of the features and then ordered according to their importance^43^. SHAP allocates each feature an importance value for a particular prediction. This SHAP feature selection approach is based on game theory^44^; SHAP values break down a prediction to show the impact of each individual feature. Suppose each feature is $x_i$, is replaced by $z_i$ for determining whether the feature value $x_i$ exists or not. SHAP represents the explanation as:

10 $$\begin{aligned} g(z) = \phi _o + \sum _{i=1}^{M} \phi _iz_i \end{aligned}$$

In the above equation, *g* represents the explanation model; $z \in {0,1} ^M$ represents the coalition vector; 0 and 1 indicate that the corresponding feature is absent or present, respectively; the number of input features included in the model is denoted as *M*; and $\phi _i \in R, \phi _i$ represents the feature attribution values for a feature *i*. Considering the game theory concept, Shapley values can be calculated using the following equation:

11 $$\begin{aligned} \phi _i = \sum _{S \subset M \setminus \{i\}} \frac{|S|!(M - |S| -1)!}{M!} [f_x(S \cup \{i\} - f_x(S))] \end{aligned}$$

In the above equation, *M* represents the set of features in the model; all feature subsets achieved from *M* are represented as *S*; the function computes the total contribution of a given features set *S*; $S \subset M \setminus \{i\}$ represents the value of the corresponding feature when *i* is known, versus when the corresponding feature value *i* is unknown for all subsets.

One of the important features of the SHAP is the barplot in the form of rectangular horizontal bars, where the length of the bars represents the importance of a given feature. As we need the global significance, we sum the contribution of each feature, or absolute Shapley values.

12 $$\begin{aligned} I_j = \sum _{i=1}^{n} |\phi _j^{(i)}| \end{aligned}$$

**Figure 2:**
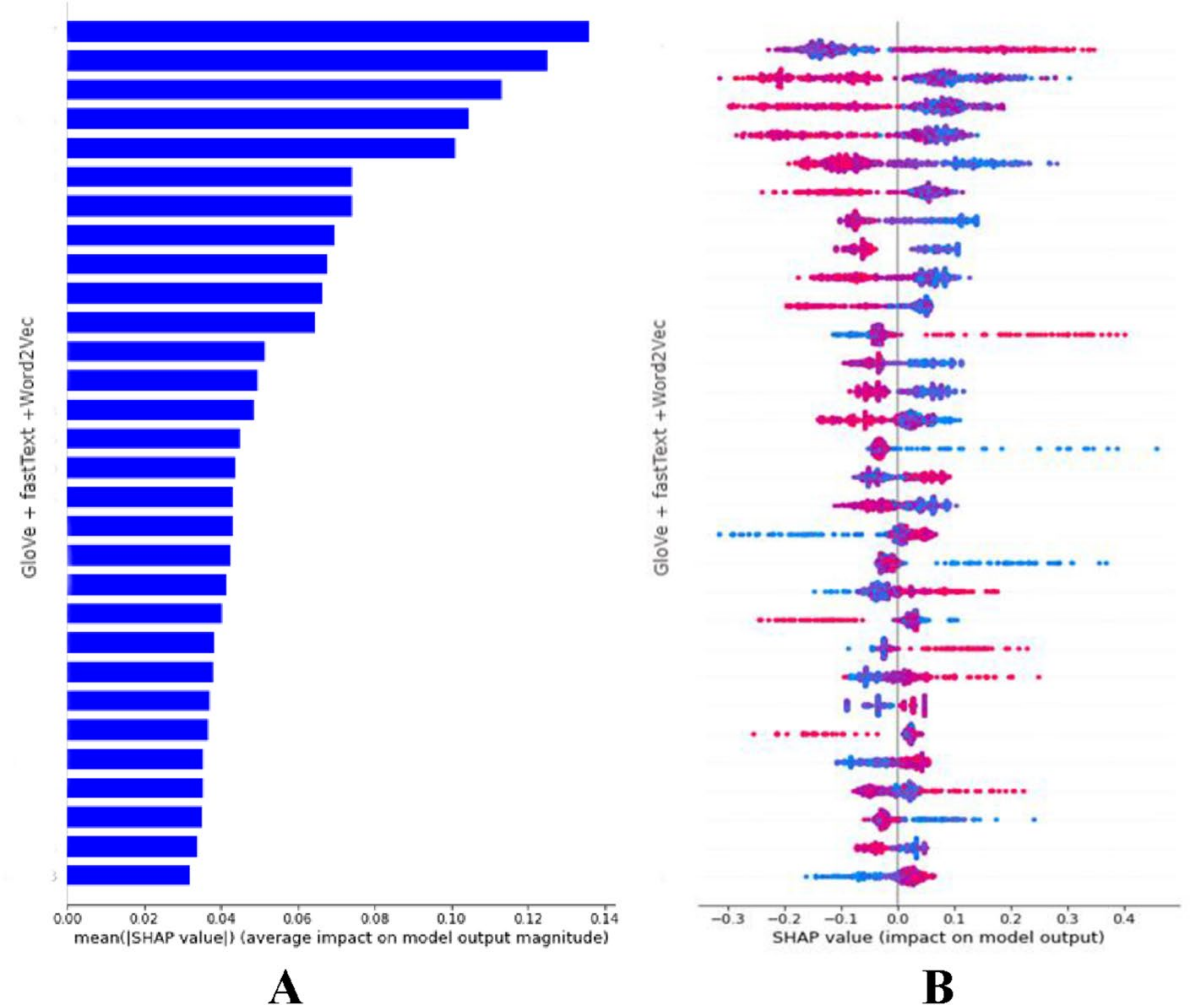
The SHAP importance bar graph results for the GloVe + fastText + Word2Vec dataset with 400 feature dimensions are presented. (**A**) bar plot generated by SHAP shows the important features in the form of horizontal bars (**B**) SHAP summary plot for the 400 feature dimensions.

Then, we plot each of the features by sorting them in decreasing order. Figure 2A shows the important features based on SHAP contributions for the XGBoost trained before predicting DBPs. The SHAP summary plot gives a high-level composite view that displays the importance of features with feature effects. Each point in the plot represents a SHAP value for a specific feature of an instance. The values that pull the prediction power of the model downwards are on the left, and the values that push the prediction further up are on the right. On the y-axis, the features are placed in descending order, and on the x-axis, there is a scale representing the Shapley value with a vertical line at point zero. The positive and negative values are to the right and left part of that vertical line, respectively. Here the colors separate the relative size of the features between instances. Specifically, low values are colored blue and high values are colored red. Overlapping more data points in the y-axis direction shows the distribution of SHAP values for each individual feature. Moreover, in the summary plot, we clearly observe the relationships between the value of a feature and the effect on the prediction. Figure 2B shows the SHAP summary plot, which orders important features for identifying DBPs.

### Implementation of convolutional neural network

CNNs are a type of deep learning model commonly used in applications including recommender systems, image and video recognition, and natural language processing^45,46^. In CNN architecture, the deeper convolutional layers (CLs) lead to learning high dimension features using sliding convolution kernels on the upper part of previous layers with different hyper-parameter settings such as filters, control layer outputs, stride, and zero-paddings. Pooling layers (PLs) are able to reduce the input feature size and offer translation invariance by local non-linear operations^45^. Fully connected layers (FCLs) utilized to classify the tasks, consisting of an equal number of output neurons as artificial neural networks.

Each neuron is completely linked to all of the nodes in the preceding and subsequent levels^47^. After adding one additional CL and max-PL to the process, the technique demonstrated a significant improvement in terms of computational complexity and program runtime. The following equation may be used to compute the outputs of each convolutional layer:

13 $$\begin{aligned} y_k^l = f\big ( \sum _{m} W_{m,k}^l y_m^{l-1} + b_k^l \big ) \end{aligned}$$

The layer index is *l*, while the input and output feature maps are *m* and *k*, respectively. Specifically, $y_k^l$ denotes the *k*th feature map of the *l* layer’s input, while $y_m^{(l-1)}$ denotes the $m-$th feature map of layer $l-1$ output. The weight tensor and bias term, respectively, are *W* and *b*. Back-propagation and adaptive estimating approaches were used to reduce cross-entropy loss^47^. Our model’s output layer is essentially a logistic regression classifier. It takes $y_k^l$ as an input and computes the following:

14 $$\begin{aligned} \hat{y} = f(W^ly^l + b^l) \end{aligned}$$

The output $\hat{y}$is the final predicted score; *W* is the weight matrix; *b* is the bias vector. Each output size is 2, denoting positive or negative classes for the binary classification task of DNA binding predictions. In order to discover suitable parameters, we want to minimize cross-entropy loss by adaptive moment estimation and back-propagation techniques:

15 $$\begin{aligned} loss = - \frac{1}{N} \sum _{i=1}^{N} y_ilog\hat{y_l} + (1-y_i)log(1-\hat{y_l}) \end{aligned}$$

To improve the model’s efficiency, batch normalization and dropout techniques^48^ were employed. The dropout in FCLs decreases by a few units during the training phase, whereas batch normalization helps to standardize the inputs into unit standard deviation and zero means. Furthermore, dropout was able to overcome the problem of overfitting, and batch normalization supported the model with sufficient learning ratios.

To achieve a better performance, hyperparameter optimization plays a vital part in the implementation of the proposed methodology. The following hyperparameters are optimized before training the model: learning rate, number of filters, kernel size, batch size, number of hidden layers, optimizers, dropout layers, and activation function. Here, three convolutional layers are used as hidden layers in the CNN model architecture. In addition, 32, 48, 64 filters and kernel sizes of 3, 4, 5 are used. Using ReLu as an activation in the hidden layers and Sigmoid in the fully connected layer results in the desired outcome. Dropout layers with dropout rates of 0.2, 0.3, and 0.5 are used to prevent overfitting. With extensive experimentation, employment of the Adam optimizer with a learning rate of 0.00001 and binary cross-entropy loss function shows the optimal result. Table 1 comprehensively illustrates the hyperparameters used in our method. Detailed parameter settings of the other three classifiers for different feature encoding are also listed in Table 6.

### Performance evaluation

The performance of Deep-WET was evaluated in terms of six standard performance metrics for the binary classification problem including accuracy (ACC), sensitivity (Sen),specificity (Spe), Matthew’s coefficient correlation (MCC), and precision (Pre).

16 $$\begin{aligned} ACC= & {} \frac{TP + TN}{TN+TP+FN+FP} \end{aligned}$$

17 $$\begin{aligned} Sen= & {} \frac{TP}{TP + FN} \end{aligned}$$

18 $$\begin{aligned} Spe= & {} \frac{TN}{FP+TN} \end{aligned}$$

19 $$\begin{aligned} MCC= & {} \frac{(TP\times TN) - (FP\times FN)}{\sqrt{(TP+FP)\times (TP+FN)\times (TN+FP)\times (TN+FN)}} \end{aligned}$$

20 $$\begin{aligned} Pre= & {} \frac{TP}{TP+FP} \end{aligned}$$

21 $$\begin{aligned} F1\, score= & {} \frac{2\times (Precision\times Recall)}{Precision + Recall} \end{aligned}$$

where *TP*, *FP*, *TN*,  and *FN* respectively represent the number of true positives (correctly classified positive), false positives (incorrectly classified as positive), true negatives (correctly classified negative), and false negatives (incorrectly classified as negative), respectively. Furthermore, the AUC metric was also used to evaluate the performances of the proposed DeepWET model, where the curve is plotted by TPR (sensitivity) and FPR (1 – specificity) with different threshold settings.

**Table 1:** Hyperparameters setting of CNN classifiers.

Hyperparameters	Range
Learning rate	[0.00001, 0.01, 0.001, 0.0001]
Number of filters	[32, 48, 64]
Kernel size	[3, 4, 5]
Batch size	[16, 32, 64, 128]
Number of hidden layers	[2, 3]
Optimizer	[‘Adam’]
Dropout rate	[0.2, 0.3, 0.5]
Activation function	[’relu’, ’sigmoid’]

### Experimental setup and packages

All tests in this study were carried out on three independent computers with the following settings, using Python version 3.7.7 or above:

- A desktop computer with Intel Core i5 CPU @ 2.71GHz x 4, Windows 10, 64-bit OS and 8 GB RAM.
- A desktop computer with Intel Core i5 CPU @ 2.11GHz x 4, Windows 10, 64-bit OS and 8 GB RAM.
- A server machine with Intel Core i5-3320M CPU @ 2.60GHz x 4, Ubuntu 18.04.2 LTS, 64-bit OS, 13 MB L3 cache and 64 GB RAM.

CNN classifier and SHAP technique were employed for model learning and feature selection on TensorFlow 2.0 and SHAP 0.39.0 Python libraries to implement them. We utilized improved parameter settings of the CNN algorithm such as batch size 16, kernel size 4, 2 hidden layers, and dropout rate 0.5. Several graphs were plotted in this experiment using Matplotlib^49^, Seaborn^50^, and Plotly^51^, in addition to pre-installed Python tools.

## Results and discussion

### Performance comparison of different feature encodings

**Figure 3:**
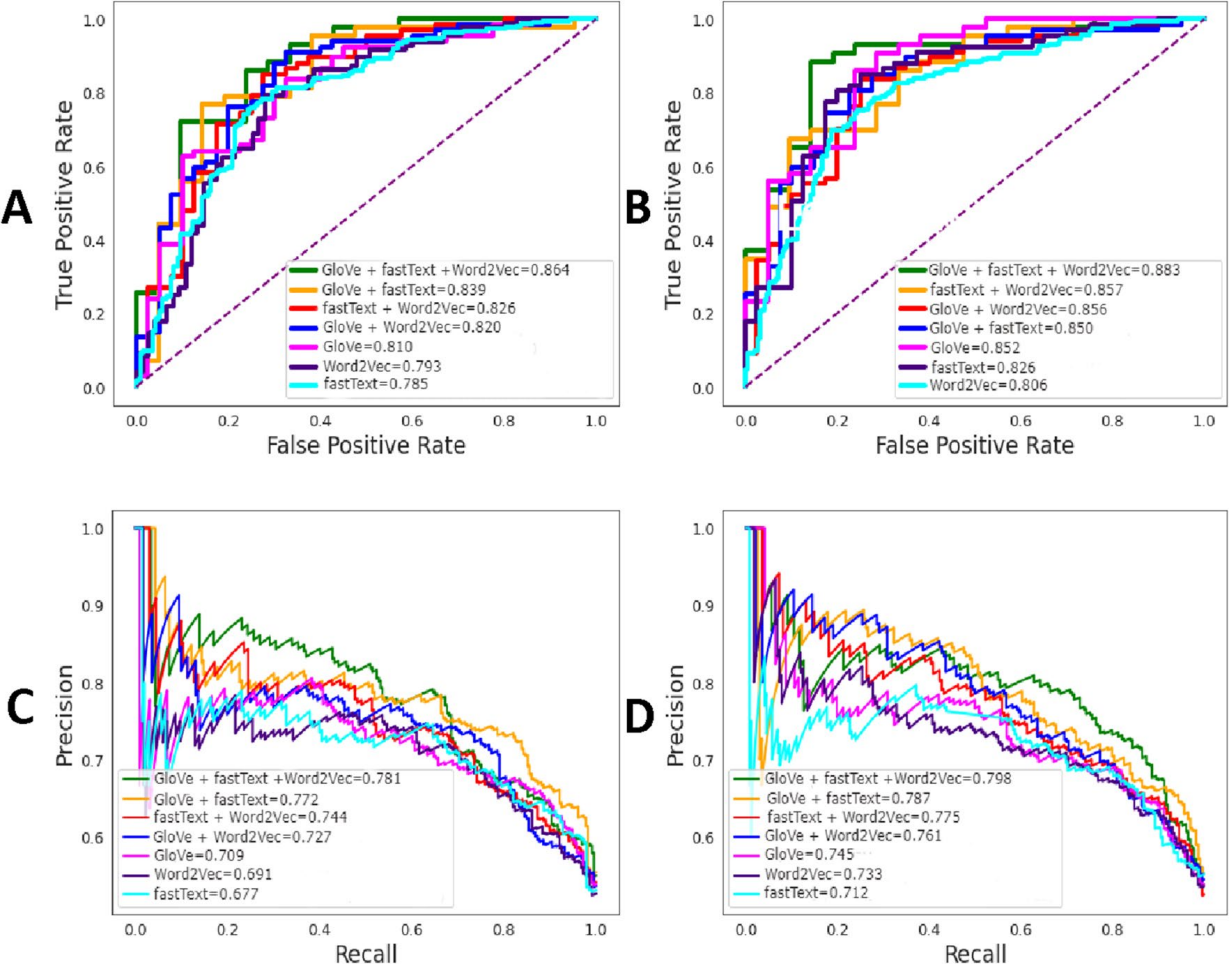
ROC curves and AUPR curves of CNN classifiers are depicted for both single and hybrid feature spaces without feature selection (**A**, **C**) and with SHAP-based feature selection (**B**, **D**).

In this section, we systematically evaluated the effect of various feature encodings, including single-feature (GloVe, fastText, and Word2Vec) and weighted-feature (GloVe + fastText, GloVe + Word2Vec, fastText + Word2Vec, and GloVe + fastText + Word2Vec) encodings in DBP identification. These features were inputted to a CNN classifier to evaluate their corresponding models using the 5-fold cross-validation test. The cross-validation performance of variant CNN classifiers trained with different features are provided in Table 2 and Fig. 3A. It is worth noting that the parameters of CNN classifiers were carefully determined to improve their performance under the 5-fold cross-validation process.

**Table 2:** Performance comparison of CNN classifiers trained with different feature encodings on the training dataset.

Feature	AUC	ACC (%)	Sen (%)	Spe (%)	MCC	Pre (%)	F1
GloVe	0.810	75.00	71.15	77.63	0.485	68.52	0.698
fastText	0.785	73.44	67.24	78.57	0.462	72.22	0.696
Word2Vec	0.793	71.09	73.13	68.85	0.420	72.06	0.726
fastText + Word2Vec	0.826	75.78	70.18	80.28	0.508	74.07	0.721
GloVe + Word2Vec	0.820	76.64	71.96	85.00	0.523	77.50	0.713
GloVe + fastText	0.839	78.12	72.96	89.19	0.549	80.95	0.738
GloVe + fastText +Word2Vec	0.864	79.07	75.10	91.49	0.585	86.21	0.740

Among single-based features, GloVe outperformed fastText and Word2Vec in terms of all performance metrics. The AUC, ACC, Sen, Spe, and MCC of GloVe were 0.810, 75.00%, 71.15%, 77.63% and 0.485, respectively. Interestingly, AUC, ACC, and MCC of GloVe were 2.5–1.7%, 1.56–3.91%, and 2.3–6.5% higher than fastText and Word2Vec, respectively. A weighted feature was created by adding different combinations of the single feature extraction methods in order to improve the predictive performance. As can be seen from Table 2, we observe that the performance the combination of GloVe, fastText and Word2Vec is better than those of other three weighted features in terms of all performance metrics. The ACC, Sen, Spe, and MCC of the combination of GloVe, fastText and Word2Vec are 79.07% 64.10%, 91.49% and 0.585, respectively, which are 0.95–3.29%, 2.14–4.92%, 2.30–11.91%, 0.036–0.077%, 5.26–12.14% and 0.002–0.027% higher than other combination features, respectively. Figure 3A shows that the AUC value of GloVe+fastText+Word2Vec 0.864, which is larger than the other three weighted features. Overall, we observed that the Sen value of individual features was slightly higher than that of the corresponding weighted features in some cases. Moreover, the performance of the top weighted features (GloVe + fastText + Word2Vec) is significantly higher than the single-view feature in terms of all evaluation metrics. Weighteds features archive higher prediction performances to the single-view feature in terms of all evaluation metrics. Therefore, in this study, the GloVe + fastText + Word2Vec feature outperformed other single and weighted features and is considered as the optimal one in termes of computational cost and predictive performance.

### Feature section approaches improve the predictive performance

The original feature subsets extracted from feature encoding techniques might contain noisy and redundant information that can affect the classifiers’ performance. Therefore, we utilized feature selection methods to determine important features from the original feature subsets. Here, three feature selection techniques, including RFE^52^, LASSO^53^, and SHAP^42^, were utilized for determining the important features from GloVe + fastText + Word2Vec feature encoding. In our experiment, we ranked all features using its importance obtained from RFE, LASSO, and SHAP and then established the six feature subsets that consisted of the top-ranked features ranging from top 200 to the top 450 features with an interval of 50. Then, for each feature selection technique, the six feature subsets were fed to develop individual CNN classifiers whose corresponding prediction results based on a 5-fold cross-validation were provided in Table 3.

**Table 3:** Performance comparison of various feature sets derived from different feature selection techniques.

Feature selection	No. of features	AUC	ACC (%)	Sen (%)	Spe (%)	MCC	Pre (%)	F1
RFE	200	0.859	76.74	58.82	88.46	0.503	76.92	0.667
	250	0.853	79.69	65.38	89.47	0.574	80.95	0.723
	300	0.866	82.55	71.87	88.89	0.621	79.31	0.754
	350	0.853	80.23	85.11	74.36	0.600	80.00	0.825
	400	0.876	81.40	67.57	91.83	0.622	86.20	0.758
	450	0.866	79.10	62.50	88.89	0.541	76.92	0.690
Lasso	200	0.837	77.57	69.05	83.07	0.526	72.50	0.707
	250	0.869	81.25	82.86	79.31	0.622	82.86	0.829
	300	0.867	81.35	87.50	76.09	0.581	76.09	0.814
	350	0.856	79.10	81.82	76.19	0.581	78.26	0.800
	400	0.882	81.40	64.71	92.30	0.607	84.61	0.733
	450	0.877	79.68	65.38	89.47	0.574	80.95	0.723
SHAP	200	0.873	76.56	60.00	91.18	0.544	85.71	0.706
	250	0.866	80.25	65.79	91.67	0.604	86.21	0.746
	300	0.883	81.31	66.66	91.89	0.616	85.70	0.750
	350	0.880	81.40	68.57	90.19	0.611	82.76	0.750
	400	**0.883**	**82.56**	**69.44**	**92.00**	**0.641**	**86.21**	**0.769**
	450	0.863	80.23	66.67	90.00	0.591	82.76	0.739

Significant values are in bold.

As seen in Table 3, the optimal subsets containing top 300, 400, and 400 optimal features derived from the RFE, LASSO and SHAP techniques, respectively, outperformed other feature sets in terms of both ACC and AUC. In the meanwhile, the performance of the optimal subsets from the SHAP technique outperformed than the RFE and LASSO techniques. To be specific, the AUC, ACC, Sen, Spe, MCC, Pre, and F1 of the optimal subset from the SHAP technique were 0.883, 82.56%, 69.44%, 92.00%, 0.641, 86.21, and 0.769, respectively. Thus, the optimal subset derived from the SHAP technique was considered to develop our proposed model. To check the effectiveness of the optimal subset, we compared its performance with the original feature set. As shown in Tables 2 and 4, the ACC, Sen, MCC and F1 of the optimal subset were 3.49%, 5.34%, 5.60%, and 3.40% higher than the original feature set. For convenience of discussion, the CNN classifier combined with the optimal subset from the SHAP technique is referred herein as Deep-WET.

Altogether, the SHAP technique was a powerful approach for implementing DNA binding protein datasets. To make a clear comparison of prediction effects, the results of the SHAP importance bar graph on the GloVe + fastText + Word2Vec dataset for 400 feature dimensions are shown in Fig. 2A. In Fig. 2A, the bar plot generated by SHAP shows the important features in the form of horizontal bars, with length representing the importance of features. We summarized the most significant features by sorting them in decreasing order based on absolute Shapley values. In addition, the SHAP summary plot for 400 feature dimensions is shown in Fig. 2B. It represents a high-level composite look that indicates the important features and effects. Each point depicts a SHAP score in the plot for a particular feature instance. Notably, we can observe the relationship between the feature value and the effect on prediction in the SHAP summary plot.

**Table 4:** Performance comparison of CNN classifiers trained with different optimal feature sets on the training dataset.

Feature	AUC	ACC (%)	Sen (%)	Spe (%)	MCC	Pre (%)	F1
GloVe	0.852	75.70	63.46	87.27	0.524	82.50	0.717
fastText	0.826	77.91	63.89	88.00	0.542	79.31	0.708
Word2Vec	0.806	74.22	66.67	81.54	0.488	77.78	0.718
fastText + Word2Vec	0.857	77.57	68.18	84.13	0.532	75.00	0.714
GloVe + Word2Vec	0.856	79.44	70.45	85.71	0.571	77.50	0.738
GloVe + fastText	0.850	80.37	69.39	89.66	0.608	85.00	0.764
GloVe + fastText +Word2Vec	0.883	82.56	69.44	92.00	0.641	86.21	0.769

From the above-mentioned observations and discussion, we concluded that the SHAP technique was a more powerful and effective feature selection one; therefore, this technique was chose for selecting a subset of features for predicting DBPs herein. In addition, we also applied the SHAP technique in other types of features whose corresponding prediction results were summarized in Table 4 and Fig. 3. By comparing the performance of the models without feature selection (Table 2 along with Fig. 3A and C) and with the SHAP-based feature selection (Table 4 along with Fig. 3B and D), the models with the SHAP-based feature selection achieve better performance than those of the models without feature selection.

### Hyperparameter of CNN

The hyperparameter learning rate controls how the model changes according to estimated error each time the weights are updated. Finding the optimal learning rate can be challenging, because a higher learning rate makes the gradient drop faster, and a lower learning rate leads to the gradient hardly converging. Here, the faster rate of gradient drop results in informative and meaningful features failing to get extracted over each iteration, and the lower rate of gradient convergence results in a longer training time. Therefore, five learning rates in a range of 1e–6 to 1e–2 are implemented for the proposed model to find the optimal performance. From Table 5, the learning rate of 0.00001 gives the highest performance compared to implementing the remaining four learning rates. However, the sensitivity value of using the 0.00001 learning rate is suboptimal compared to the value of the 0.0001 and 0.001 learning rates.

**Table 5:** Cross-validation results of CNN classifiers trained with different learning rates.

Learning rate	AUC	ACC (%)	Sen (%)	Spe (%)	MCC	Pre (%)	F1
1e–2	0.835	79.06	64.29	86.21	0.515	69.23	0.667
1e–3	0.803	81.39	**72.73**	84.38	0.543	61.54	0.667
1e–4	0.838	81.25	69.57	87.80	0.586	76.19	0.727
1e–5	0.883	82.56	69.44	92.00	0.641	86.21	0.769
1e–6	0.874	79.07	61.11	92.00	0.571	84.62	0.710

Significant values are in bold.

### Comparison of Deep-WET with conventional ML classifiers

**Figure 4:**
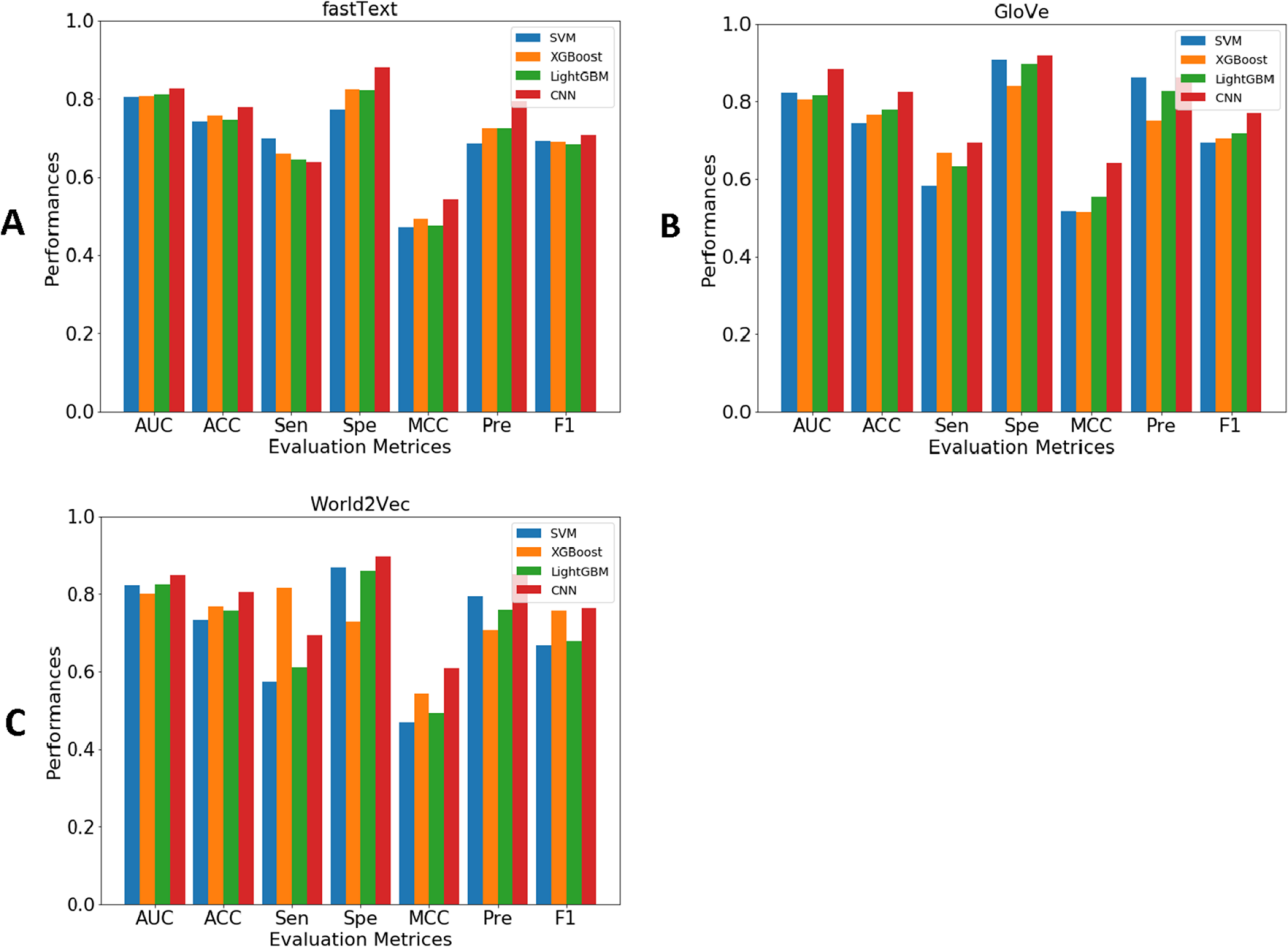
Performance comparison of three feature groups for SVM, XGBoost, LightGBM, and CNN classifiers under 5-fold cross-validation test on various evaluation metrics: (**A**) GloVE, (**B**) Word2Vec, and (**C**) fastText.

**Figure 5:**
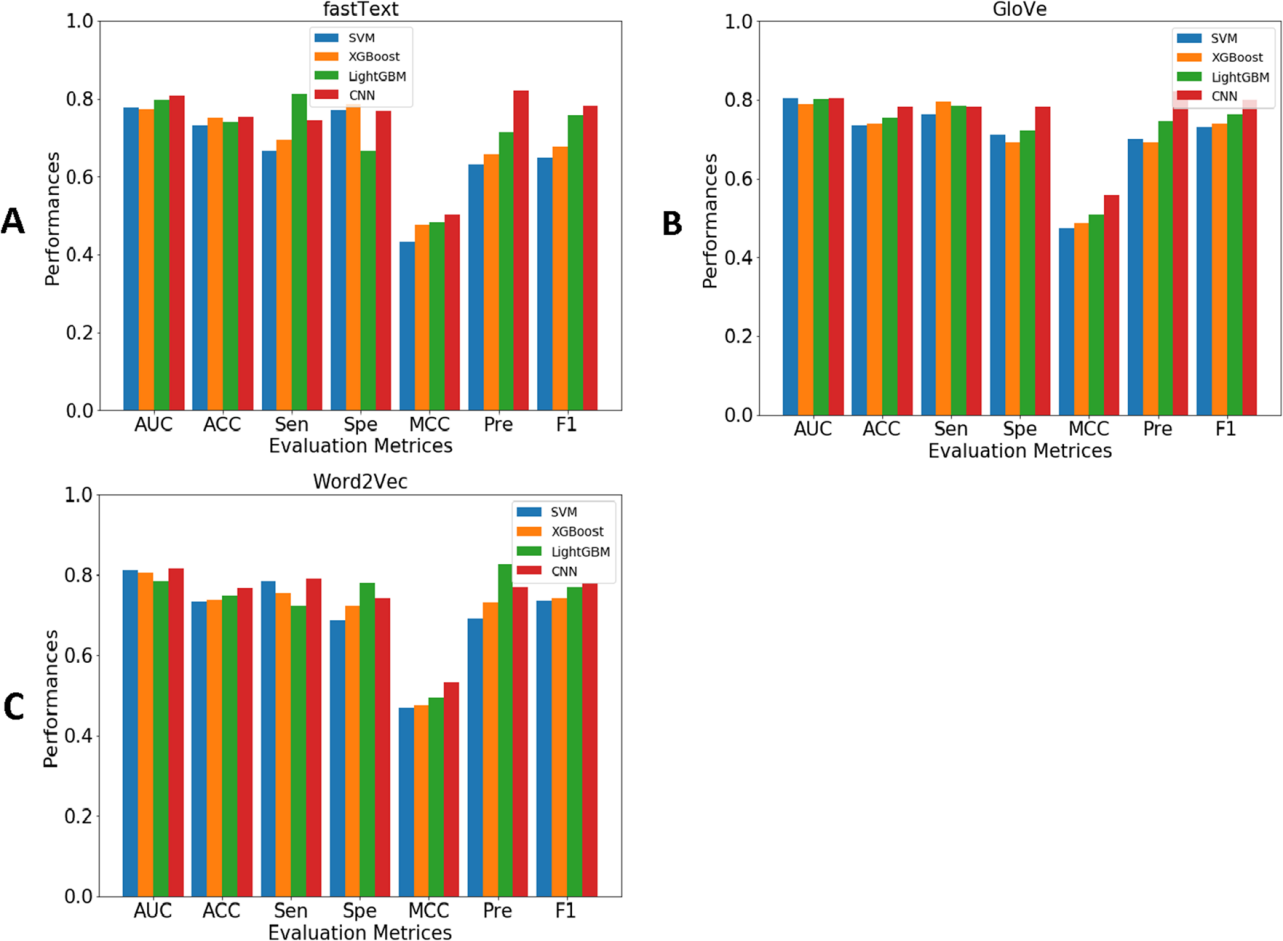
Performance comparison of three feature groups for SVM, XGBoost, LightGBM, and CNN classifiers under independent test on various evaluation metrics: (**A**) GloVE, (**B**) Word2Vec, and (**C**) fastText.

**Figure 6:**
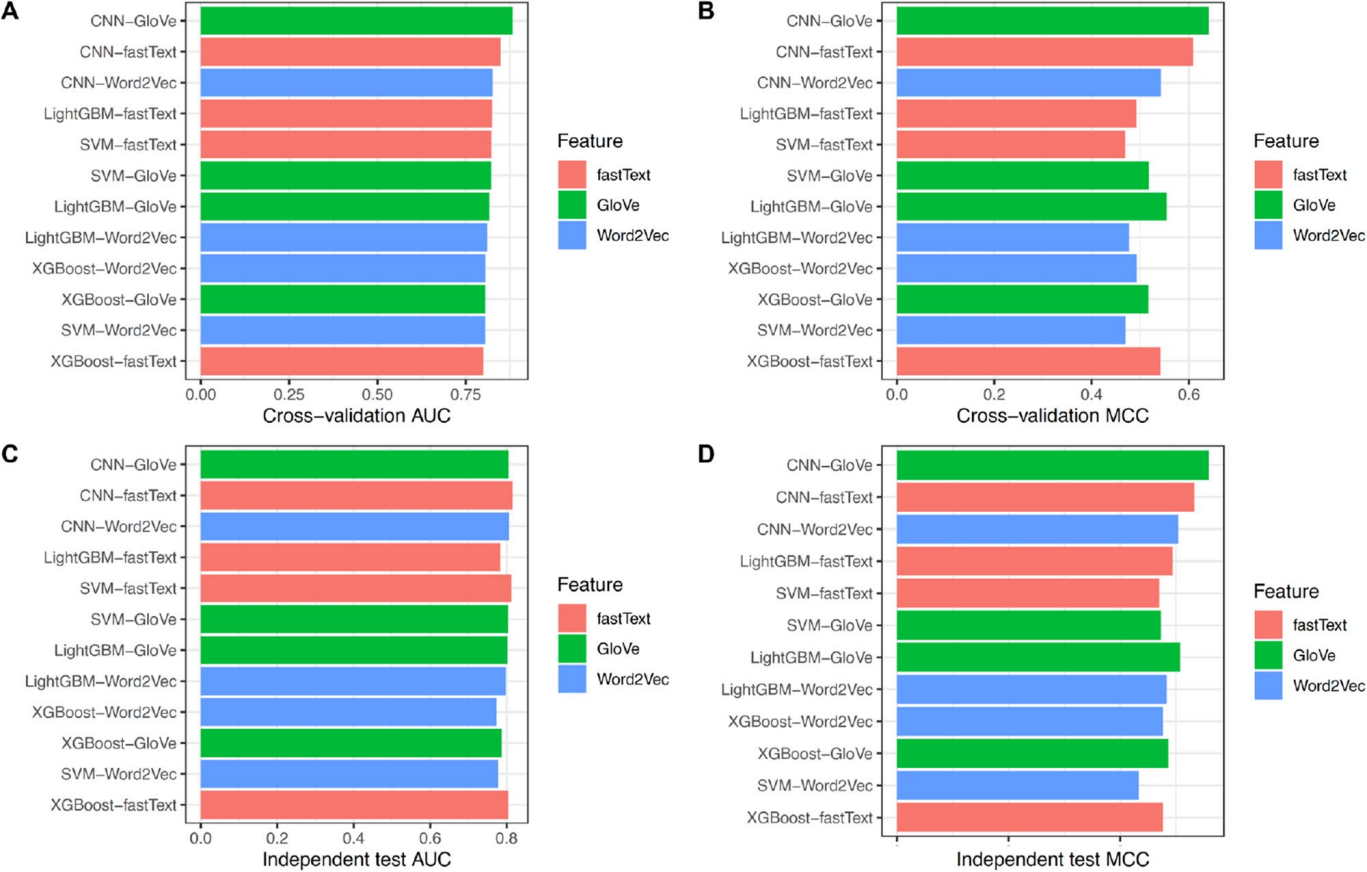
Performance comparison of various machine learning classifiers trained with three feature groups, utilizing different classifier and feature representations, is presented in terms of AUC and MCC evaluation metrics under 5-fold cross-validation (**A**, **B**) and independent testing (**C**, **D**).

To evaluate the performance of the proposed Deep-WET, we compared its predictive performance with conventional ML classifiers. Herein, the conventional ML classifiers were built using four well-known ML classifiers (i.e., SVM^40^, XGBoost^41^, LightGBM^44^, and CNN^54^) and the three NLP-based word embedding techniques (i.e., GloVe, fastText, and Word2Vec). In total, 11 conventional ML classifiers were created in this study. It is noteworthy that the parameters of all ML classifiers were carefully optimized to improve their prediction capability under a 5-fold cross-validation procedure. In these experiments, classifiers have been trained a total of 24 times. The prediction performance based on both 5-fold cross-validation and independent tests are listed in Tables 7-8. In addition, their respective graphs are shown in Figs. 4, 5, 6.

**Table 6:** Cross-validation results of different ML classifiers and feature encoding schemes.

Feature	Classifier	AUC	ACC (%)	Sen (%)	Spe (%)	MCC	Pre (%)	F1
GloVe	SVM	0.822	74.42	58.14	90.69	0.517	86.21	0.694
	XGBoost	0.805	76.63	66.67	83.87	0.516	75.00	0.706
	LightGBM	0.817	77.90	63.16	89.58	0.554	82.76	0.716
	CNN (Deep-WET)	**0.883**	**82.56**	**69.44**	**92.00**	**0.641**	**86.21**	**0.769**
fastText	SVM	0.823	73.26	57.50	86.96	0.469	79.31	0.667
	XGBoost	0.800	76.74	81.58	72.92	0.541	70.45	0.756
	LightGBM	0.825	75.58	61.11	86.00	0.492	75.86	0.677
	CNN	0.849	80.37	69.39	89.66	0.608	85.00	0.764
Word2Vec	SVM	0.805	74.21	69.81	77.33	0.470	68.52	0.692
	XGBoost	0.806	75.69	65.91	82.54	0.493	72.50	0.691
	LightGBM	0.810	74.77	64.44	82.26	0.477	72.50	0.683
	CNN	0.826	77.91	63.89	88.00	0.542	79.31	0.708

Significant values are in bold.

As can be seen from Table 6, Deep-WET achieved the overall best performance compared with the compared ML classifiers in terms of almost performance metrics, with the only exception of the Sen. Meanwhile, CNN-fastText and CNN-Word2Vec were the second-best and third-best classifiers in terms of ACC. To be specific, the ACC values of Deep-WET, CNN-fastText, and CNN-Word2Vec were 82.56%, 80.37%, and 77.91%, respectively. In addition, Deep-WET’s AUC, ACC, Spe, and MCC were 3.40%, 2.19%, 2.34% and 3.30%, respectively, higher than the second-best method CNN-fastText. In case of the independent test results, Deep-WET still outperformed the compared ML classifiers in terms of ACC, Spe, MCC, Pre and F1. Deep-WET’s ACC, Spe, MCC, Pre, and F1 were 1.37%, 3.84%, 2.60%, 5.13% and 2.10%, respectively, higher than the second-best method CNN-fastText.

**Table 7:** Independent test results of different ML classifiers and feature encoding schemes.

Feature	Classifier	AUC	ACC (%)	Sen (%)	Spe (%)	MCC	Pre (%)	F1
GloVe	SVM	0.803	73.52	76.25	71.11	0.473	70.11	0.731
	XGBoost	0.787	73.97	79.41	69.23	0.486	69.23	0.740
	LightGBM	0.802	75.34	78.37	72.22	0.507	74.36	0.763
	CNN (Deep-WET)	0.805	78.08	78.05	78.13	0.559	82.05	0.800
fastText	SVM	0.812	73.20	78.26	68.63	0.470	69.23	0.735
	XGBoost	0.804	73.77	75.41	72.13	0.476	73.02	0.742
	LightGBM	0.783	74.59	72.22	78.00	0.494	82.54	0.770
	CNN	**0.816**	76.71	78.95	74.29	0.533	76.92	0.779
Word2Vec	SVM	0.778	73.19	66.67	77.05	0.433	63.16	0.649
	XGBoost	0.773	75.25	69.44	78.69	0.476	65.79	0.676
	LightGBM	0.797	73.98	81.08	66.67	0.483	71.43	0.760
	CNN	0.807	75.35	74.42	76.67	0.504	82.05	0.781

Significant values are in bold.

To further the comparison, Figs. 4, 5 and 6 illustrate the cross-validation and independent test performance for our proposed Deep-WET approach along with four robust classifiers with all the evaluation metrics. From Tables 7, 8 and Figs. 4, 5 and 6, we can summarize several observations as follows: (i) GloVe features obtained the highest predictive results as compared to fastText and Word2Vec; however, these three feature-encoding techniques all achieved promising performance for the CNN classifiers, followed by LightGBM, XGBoost, and SVM classifier. Word2Vec achieved relatively lower performance, whereas fastText was slightly better than Word2Vec, (ii) CNN classifier consistently achieved the highest results compared to the other three classifiers for all three feature-encoding techniques, and (iii) Finally, our proposed Deep-WET achieved better performance than other conventional ML classifiers, highlighting its superior discriminative power.

### Comparison of Deep-WET with the state-of-the-art methods

To further validate the discriminative power of Deep-WET method, we compared its prediction performance against other existing DBP methods, including DPP-PseAAC^10^, PseDNA-Pro^19^, iDNA-Prot^18^, iDNA-Prot|dis^14^, PSFM-DBT^22^, Local-DPP^11^, HMMBinder^23^, iDNAProt-ES^7^, IKP-DBPPred^24^ Xiuquan et al.^55^, iDRBP-MMC^56^ and TargetDBP^25^, on the independent test data. The prediction performance of the existing methods were obtained by submitting protein sequences in the independent test dataset (148 DBPs and 148 non-DBPs) to their own webservers. Since the web sever of iDNAProt-ES was not functional, the prediction results of iDNAProt-ES were obtained from the reimplementation of iDNAProt-ES and the standalone version of HMMBinder^23^, respectively. Table 8 shows the prediction results of Deep-WET and other existing methods.

**Table 8:** Performance comparisons of DeepWET with the state-of-the-art methods on the independent test dataset.

Predictor^a^	AUC	Sen (%)	Spe (%)	MCC	Pre (%)	F1
DPP-PseAAC	61.15	55.41	66.89	0.225	62.60	0.588
iDNA-Prot	62.16	63.51	60.81	0.243	61.84	0.627
iDNA-Prot|dis	68.24	72.30	64.19	0.366	66.88	0.695
PseDNA-Pro	67.23	78.38	56.08	0.354	64.09	0.705
PSFM-DBT	68.58	71 .62	65.54	0.372	67.52	0.695
IKP-DBPPred	58.11	52.70	63.51	0.163	59.09	0.557
Local-DPP	48.65	3.38	**93.92**	– 0.06	35.71	0.062
iDNAProt-ES(on PDB1075)	71.62	91 .89	51.35	0.473	65.38	0.764
TargetDBP	76.69	76.35	77.03	0.534	76.87	0.766
Xiuquan et al.	77	–	–	–	–	–
iDRBP-MMC	70	–	–	–	–	–
Deep-WET	**78.08**	**78.05**	**78.13**	**0.559**	**82.05**	**0.800**

Significant values are in bold.

^a^The prediction performance of the existing methods were obtained by submitting protein sequences in the independent test dataset (148 DBPs and 148 non-DBPs) to their own webservers.

According to the F1 and MCC values, these two evaluation metrics of binary predictions, recorded in Table 8, we can see that Deep-WET has superior performance over other exiting methods in terms of ACC, MCC, Pre, and F1. Notably, by comparing the proposed Deep-WET approach with the second-best predictor TargetDBP in terms of ACC, we observe that Deep-WET achieved improvements of 1.39%, 1.70%, 2.50%, 5.18%, and 3.40% on ACC, Sen, MCC, Pre, and F1, respectively. Although, iDNAProt-ES obtained the highest Sen value of 91.89%, this method provided the lowest Spe value of 51.35%. The main reason behind this high Sen is that iDNAProt-ES has lower false negative (FN) prediction. In contrast, Local-DPP obtained the highest prediction performance in terms of Spe (93.92%) and shows much lower Pre scores (35.71%) producing many false negatives (FN) during prediction, but Acc values of iDNAProt-ES and Local-DPP are lower than those of Deep-WET. Taken together, these results demonstrated that Deep-WET has a great potential for DBP prediction.

### Ablation study

Our CNN model has key components such as convolutional filters, pooling strategies, kernel sizes and fully connected layers, etc. Here, we have conducted ablation studies using the GloVe + fastText + Word2Vec dataset under 5-fold CV, assessing how each individual component influences the predictive performance of Deep-WET:

- Remove Specific Convolutional Filters (RSCF): we removed specific filters in the convolutional layers responsible for capturing sequence motifs or patterns linked to DNA binding.
- Variation in Pooling Strategies (VPS): adjust the baseline model by altering pooling strategies to evaluate how these changes affect the recognition of relevant sequence features.
- Variation in Kernel Sizes (VKS): Explore diverse kernel sizes within the convolutional layers to capture sequence motifs associated with DNA binding of different lengths.
- Removal of Fully Connected Layers (RFCL): create a modified version of the baseline model by removing one or more fully connected layers to examine the significance of global features in the classification task.

**Figure 7:**
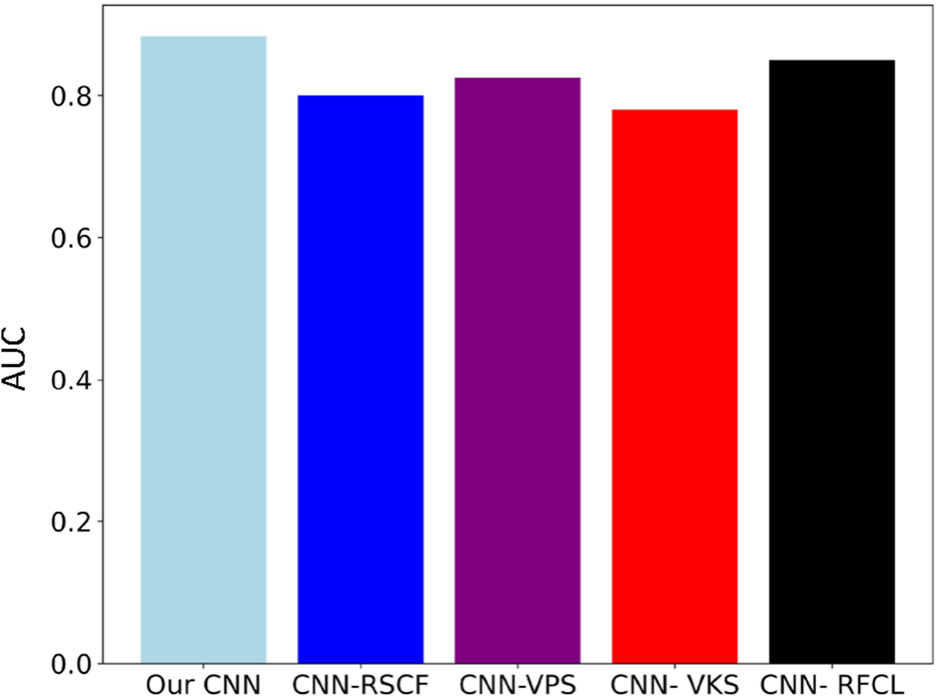
Comparative analysis between Our CNN and its ablation experiments on the GloVe + fastText + Word2Vec dataset.

Figure 7 show the performance comparison of Deep-WET and its four variants in terms of AUC on GloVe + fastText + Word2Vec dataset. We can observe that our CNN has better performance than CNN-RSCF, CNN-VPS, CNN-VKS and CNN-RFCL on experiment datasets Here, our-CNN obtains the best AUC score of 0.883, and it is 0.085%, 0.135%, 0.105% and 0.165% higher than that of CNN-RSCF, CNN-VPS, CNN- VKS and CNN-RFCL, respectively, which can illustrate that these parts in our design can improve the predictive performance. Among them, CNN-VPS and CNN-RFCL have the lowest performance. This shows that it is very important to perform the hyperparameters setting of CNN classifiers (see Table 1), that can effectively improve the performance of CNN.

### Web server, data and software availability

We used Apache (2.4.48), Python (3.8.0), and Laravel (8.16.1) to develop a web server for Deep-WET at https://deepwet-dna.000webhostapp.com/. Users can upload or input DNA binding protein sequences of viruses and humans in FASTA format to predict DBPs with probability scores. After clicking the submit button, the server will evaluate the protein sequence and check the format for processing. Prediction results will be generated in a tabular format with detailed information on the word serial number and predicted probability of DBPs and predicted class (DBPs/non-DBPs). Detailed instructions for the webserver can be found on the README option. After the final job, users will get a job ID to be used for further queries. The Deep-WET web server application stores this job ID for fifteen days. Deep-WET may have a long computational time when users input large protein sequences files, since Deep-WET needs to perform NLP-based word embedding packages to generate discriminative features and fix the suitable parameters for the CNN classifier to predict. We strongly suggest inputting a low number of DBP sequences at a time. The experimental datasets for this study are available at: https://deepwet-dna.000webhostapp.com/data.

## Conclusions

Identifying DBPs is vital to discovering fundamental protein-DNA mechanisms and understanding their biological interactions. Here, we develop a new deep learning-based approach termed Deep-WET,to achieve more accurate and improved prediction of DBPs. In Deep-WET, we extracted three NLP-based word embedding features to generate single features then combined them sequentially, and assigned weights learned through the use of the DE algorithm. The SHAP technique was utilized to gain the effective feature subsets, and a deep learning-based CNN algorithm was used as a model classifier for predicting DBPs. Comparative analysis on the independent test dataset showed that Deep-WET achieved improved performance compared with conventional ML classifiers and the existing methods, highlight the effectiveness and robustnessof the proposed Deep-WET. The improved performance of Deep-WET is mainly due to the utilization of NLP-based word embedding features that can effectively capture the characteristics of DBPs. A user-friendly web server for Deep-WET is available at https://deepwet-dna.royalit.agency/. Deep-WET is anticipated to be a powerful tool to serve the community-wide effort for the accurate and large-scale identification of potential DBPs from sequences information. Deep learning shows advanced prediction abilities in various fields of computational biology such as hERG blockers^57^ , disease-related metabolites^58,59^, single-cell^60^ and human lncRNA-miRNA interactions^61,62^. Most studies propose deep learning based models^63^ for prediction tasks. To enhance the predictive capabilities of our Deep-WET model, our future efforts will focus on three key areas: (1) although our NLP-based feature extraction is now commonly used for extracting distinct features, there may be some limitations, such as ambiguities, lexical gaps, and structural gaps. It would be interesting to use deep learning-based autoencoders^59,60,64^ to effectively convey the hidden information within DBPs sequences; (2) implementing a small-loss approach and integrating probabilistic local outlier factor (pLOF) with the extracted features to tackle the challenge of label noise in the dataset, ensuring a trustworthy application; (3) developing a graph-based deep learning model for predicting DBPs with unknown structures.

Cellular death is a fundamental and complex biological process that is an underlying driver for many diseases. Authors in^65,66^, worked for cell death. Our CNN model can be used to classify cells undergoing cell death. This deep learning network has the ability to highly predict cell death. Finally, it is possible to provide a simple Python tool that can be broadly used to detect cell death. Furthermore, our CNN model can recommend specific drugs for the disease.

## Data Availability

All the data used in this study are available at https://deepwet-dna.monarcatechnical.com/data.
